# Pregnancy-Related Anxiety and Impact of Social Media Among Pregnant Women Attending Primary Health Care

**DOI:** 10.7759/cureus.20081

**Published:** 2021-12-01

**Authors:** Hussain A Al Ghadeer, Nihad A Al Kishi, Duaa M Almubarak, Zainab Almurayhil, Fatimah Alhafith, Bayan Abduljaleel Al Makainah, Kholoud H Algurini, May M Aljumah, Maria M Busaleh, Nouh A Altaweel, Mohammed H Alamer

**Affiliations:** 1 Pediatrics, Maternity and Children Hospital, Al-Ahsa, SAU; 2 Obstetrics and Gynaecology, Maternity and Children Hospital, Al-Ahsa, SAU; 3 Obstetrics and Gynaecology, King Faisal University, Al-Ahsa, SAU; 4 Family Medicine, Al-Ahsa Health Cluster, Al-Ahsa, SAU

**Keywords:** al-ahsa, saudi arabia, social media, pregnancy, anxiety

## Abstract

Background

Pregnant women go through physiological as well as psychological changes during pregnancy. Antenatal anxiety disorders are common, with proven adverse maternal and fetal outcomes. Anxiety increases the risks for prematurity and neurodevelopmental disorders. This study aimed to estimate the prevalence of pregnancy-related anxiety and the impact of social media among pregnant women in Al-Ahsa, Saudi Arabia.

Materials and methods

This observational cross-sectional study included pregnant women who were attending antenatal care (ANC) in primary healthcare centers between May and October of 2021 in Al-Ahsa, Saudi Arabia. For data collection, a structured self-administered questionnaire was distributed randomly to eligible pregnant women. The presence of pregnancy-related anxiety was assessed by using the 10-item Pregnancy-Related Anxiety Questionnaire-Revised (PRAQ-R), Arabic version. The impact of social media was measured through Social Media Engagement Questionnaire (SMEQ).

Results

Out of 823 pregnant women, 382 were eligible. Their mean age was 26.1 ± 10.9 years. Most of them (70.4%) had adhered to ANC. However, 32.1% had a history of miscarriage, and 6.7% had previous birth with congenital anomalies. The mean scores of pregnancy-related anxiety domains were 10.6 out of 15 for fear of giving birth, 8.7 for concern of own appearance, and 6.4 out of 12 for worries about bearing a handicapped child. More than half of the participants scored 28 out of 50 for pregnancy-related anxiety. The factors that were significantly associated with pregnancy-related anxiety were healthcare workers, first trimester, and unplanned pregnancy (P < 0.05). Social media engagement showed no correlation with anxiety.

Conclusions

The pregnancy-related anxiety level was average among pregnant women in Al-Ahsa, and fear of giving birth was the most common reason. Its predictors included early pregnancy, being a healthcare provider, and unplanned pregnancy. Pregnancy-related anxiety should be diagnosed early during routine ANC for better maternal and fetal outcomes.

## Introduction

Pregnancy is the most important and challenging period among women willing to raise a child [1]. Pregnant women experience several physical changes such as weight gain and changes in body appearance; psychological changes such as stress, anxiety, and depression; and also hormonal changes [2]. Therefore, women are recommended to prepare themselves physically and mentally so they can go through such a critical and life-changing period safely without facing any complications. Such complications include miscarriage, low birth weight, impaired cognitive and physical fetal development, and possibly death [3-5]. Nonetheless, following a healthy lifestyle and maintaining mental well-being and peace of mind can make pregnancy a positive and rewarding experience [1,6]. In fact, social media can greatly influence such an important period of life for many women either positively or negatively.

Social media is a huge internet-based platform that offers fast digital communication and widespread information, images, and video clips, which can all be easily and quickly accessed through smartphones, among internet users [6]. In Saudi Arabia, the number of social media users is increasing by 2.1 million annually. In addition, approximately 80% of the total population in Saudi Arabia uses social media [7]. Pregnant women do use social media for several purposes, such as emotional support, social support, and improvement in health literacy, especially information related to pregnancy. They can certainly benefit from social media usage. One of the benefits of social media is the provision of social support, which can be greatly helpful for preventing pregnancy-related anxiety and maintaining a positive mood throughout pregnancy [8]. Conversely, social media can negatively impact the physical and psychological well-being of a pregnant woman, especially when the information is from unreliable sources and not provided by a healthcare practitioner. Furthermore, the vast amount of information from social media sometimes may have opposing views; hence, with constant viewing, it leads to a sense of mistrust, which can trigger anxiety and mental stress [9].

Therefore, the pregnancy period is the most sensitive stage in women’s lives, and pregnant women are highly at risk of developing psychological changes, which may lead to unfavorable consequences, such as preterm delivery, low birth weight, and postpartum depression [10,11]. In Saudi Arabia, the most prevalent psychological disorders during pregnancy are anxiety and depression, accounting for 23.6% and 26.8%, respectively. Unemployed women, as well as those with an unplanned pregnancy and recurrent miscarriage, have a high risk for depression and anxiety during pregnancy [12].

Hence, pregnancy-related anxiety is among the most prevalent mental conditions. This condition refers to an undesirable emotional state associated with multiple worries about childbirth, the baby’s health, and healthcare experience during pregnancy and birth [13]. Anxiety symptoms such as poor sleep and tiredness are common during pregnancy, and up to 54% of pregnant women are at risk for these symptoms [13,14]. In Kuwait, the prevalence of pregnancy-related anxiety was 15% [15], whereas in India, it was 55%, which is considered high, especially before the gestational age of 24 weeks [16]. Pregnancy-related anxiety mimics many conditions; thus, this diagnosis may be overlooked, leading to negative pregnancy outcomes such as prolonged labor, preterm delivery, and cesarean section. Therefore, screening and early detection of anxiety are critical during pregnancy to prevent complications [17]. The Pregnancy-Related Anxiety Questionnaire-Revised 2 (PRAQ-R2) is a valid assessment scale used for detecting pregnancy-related anxiety for both nulliparous and parous women [18]. Accordingly, measuring the prevalence of pregnancy-related anxiety and assessing its relationship with social media addiction in Al-Ahsa, the eastern province of Saudi Arabia, are crucial to take preventive measures in the future.

## Materials and methods

Aim

This study aimed to explore the psychological impacts of pregnancy and social media influence among pregnant women attending primary health care in Al-Ahsa, Saudi Arabia.

Study design and participants

This descriptive cross-sectional study was conducted in primary healthcare centers in Al-Ahsa, Saudi Arabia, between May and October of 2021. Any pregnant women (primigravida or multigravida) aged above 18 years and not diagnosed recently or previously with comorbidities or psychological diseases were included. Any pregnant women aged less than 18 years old and diagnosed with psychological illness were excluded from the research.

Data collection instrument and procedures

Data were collected at the primary healthcare centers. All participants provided informed consent and had autonomy for rejection. The privacy and confidentiality of results were maintained. The Institutional Review Board of King Fahad Hospital-Hofuf in Al-Ahsa approved this study (NO: 34-EP-2021). The participants were requested to fill an online or paper-based questionnaire. The survey was composed of four sections. The first section is about patient demographics (age, gender, and education level), the second section is about obstetric history (gestational age, regular follow-up on antenatal care [ANC], and history of abortion or congenital anomalies), the third section is about pregnancy-related anxiety using the 10-item PRAQ-R, Arabic version [19,20], and the fourth section is about social media engagement using the Arabic version of the Social Media Engagement Questionnaire (SMEQ) [21,22]. Each item in the PRAQ-R is answered using a five-point Likert scale (from 1 as “definitely not true” to 5 as “definitely true”) for measuring three subscales: fear of giving birth (three questions); worries about bearing a physically or mentally handicapped child (four questions); and concern of own appearance (three questions). Meanwhile, the SMEQ is a brief, valid, and effective psychometric instrument used for assessing the levels of personal use of social media by measuring the extent to which people’s key daily activities tend to involve social media. It consists of five items that measure social media usage patterns throughout different parts of the day. These items were rated on a seven-point scale, with 0 as “never” and 7 as “seven times.”

Data analysis

The data were extracted, revised, coded, and fed to the statistical software IBM SPSS, version 22 (SPSS Inc. Chicago, IL). All statistical analyses were conducted using two-tailed tests. A P-value of less than 0.05 was considered statistically significant. The total and subscale scores of PRAQ-R2 were calculated. The PRAQ-R2 has 10 items, and the subscale scores are 3-15 for fear of giving birth (F1, three items), 4-20 for worries about bearing a physically or mentally handicapped child (F2, four items), and 3-15 for concern about own appearance (F3, three items). The overall score ranges from 10 to 50. Mean scores with standard deviation and score percentage of the total were calculated for each factor and overall. As for SMEQ, an individual's composite mean days of use for each item was calculated as the range and standard deviation of the overall engagement days per week. The different factors associated with pregnancy-related anxiety levels were assessed by multivariable analysis, and the significance was determined by one-way ANOVA and independent samples t-test. The relationship between pregnant women’s anxiety score and their average number of social media engagement days per week was evaluated by correlation analysis with scatter distribution.

## Results

A total of 382 pregnant women met the inclusion criteria and completed the study questionnaire. Their ages ranged from 18 to 46 years, with a mean age of 26.1 ± 10.9 years. Slightly more than half of them (228, 59.7%) were university graduates or postgraduates. Regarding occupation, most of them were housewives (281, 73.6%). In addition, 58 (15.2%) were non-healthcare workers, whereas 43 (11.3%) were healthcare workers. Majority had a monthly income of 5,000-10,000 SR (197, 51.6%), followed by below 5,000 SR (113, 29.6%) and above 20,000 SR (9, 2.4%) (Table 1).

**Table 1:** Socio-demographic data of pregnant females, Al-Ahsa, Saudi Arabia.

Socio-demographic data	No	%
Age in years		
18-25	101	26.4%
26-30	140	36.6%
31-39	123	32.2%
40+	18	4.7%
Educational level		
Below university	154	40.3%
University/above	228	59.7%
Job title		
Housewife	281	73.6%
Non-healthcare worker	58	15.2%
Healthcare worker	43	11.3%
Monthly income		
<5,000 SR	113	29.6%
5,000-10,000 SR	197	51.6%
10,000-20,000 SR	63	16.5%
>20,000 SR	9	2.4%

Regarding obstetric history (Table 2), 83 (21.7%) of the participants were primigravida. Additionally, 101 (26.4%) were in their first trimester, and 139 (36.4%) were in their third trimester. The majority (269, 70.4%) of the participants reported being always adherent to ANC appointments, whereas only one reported never going to ANC visits. In the current pregnancy, 71 (18.6%) had complications, and only two used in vitro fertilization (IVF). The current pregnancy was previously planned among 203 (53.1%) females. Furthermore, 89 (29.8%), 96 (32.1%), and 20 (6.7%) participants reportedly had a previous pregnancy or childbirth complications, a history of miscarriage or stillbirth, and a history of giving birth with congenital anomalies, respectively.

**Table 2:** Obstetric history among pregnant females, Al-Ahsa, Saudi Arabia.

Obstetric history	No	%
Is this being your first pregnancy?		
Yes	83	21.7%
No	299	78.3%
Gestational age of the pregnancy		
First trimester (1-13 weeks)	101	26.4%
Second trimester (14-26 weeks)	142	37.2%
Third trimester (27-40 weeks)	139	36.4%
Do you adhere to your appointments in antenatal care?		
Always	269	70.4%
Often	77	20.2%
Sometimes	35	9.2%
Never	1	.3%
Do you have complications in your current pregnancy?		
Yes	71	18.6%
No	311	81.4%
Have you used in vitro fertilization (IVF) for the current pregnancy?		
Yes	2	.5%
No	380	99.5%
Is this current pregnancy previously planned?		
Yes	203	53.1%
No	179	46.9%
Have you ever had any complications during pregnancy or childbirth?		
Yes	89	29.8%
No	210	70.2%
Have you ever had a miscarriage or stillbirth?		
Yes	96	32.1%
No	203	67.9%
Have you ever had congenital anomalies?		
Yes	20	6.7%
No	279	93.3%

The results of PRAQ-R2 are presented in Table 3. Concerning the fear of giving birth, most of the participants (92.7%) were worried about the pain of contractions and the pain during delivery, which was reasonable to very relevant among 69.4% of them. The majority (92.4%) of participants also worried about delivery, which was relevant among 66.8%. Additionally, 75.4% were worried about not being able to control themselves during labor and afraid that they would scream. Regarding worries about bearing a handicapped child, 59.2% sometimes thought that their baby would be in poor health or prone to illnesses; similar results were obtained regarding fear of physical defect or physical limitation in the newborn (46.3%), fear of stillborn or infant death during or immediately after delivery (44.5%), and fear of mental disability or brain damage (43.7%). With regard to concern about their own appearance, they were mostly worried that they shall not regain their pre-pregnancy figure after delivery (80.4%), followed by worry about gaining an enormous weight (72%) and worry about their unattractive appearance (63.1%).

**Table 3:** Results of self-reported 10 items Pregnancy-Related Anxiety Questionnaire-Revised 2 (PRAQ-R2).

Domain	Items	Absolutely not relevant		Hardly ever relevant		Sometimes relevant		Reasonably relevant		Very relevant	
		No	%	No	%	No	%	No	%	No	%
Fear of giving birth	I am anxious about the delivery.	29	7.6%	13	3.4%	85	22.3%	176	46.1%	79	20.7%
	I am worried about the pain of contractions and the pain during delivery.	28	7.3%	25	6.5%	68	17.8%	129	33.8%	132	34.6%
	I am worried about not being able to control myself during labor and fear that I will scream.	94	24.6%	36	9.4%	64	16.8%	108	28.3%	80	20.9%
Worries about bearing a handicapped child	I sometimes think that our child will be in poor health or will be prone to illnesses.	156	40.8%	49	12.8%	86	22.5%	65	17.0%	26	6.8%
	I am afraid the baby will be mentally handicapped or will suffer from brain damage.	215	56.3%	30	7.9%	65	17.0%	41	10.7%	31	8.1%
	I am afraid our baby will be stillborn or will die during or immediately after delivery.	212	55.5%	36	9.4%	67	17.5%	40	10.5%	27	7.1%
	I am afraid that our baby will suffer from a physical defect or worry that something will be physically wrong with the baby.	205	53.7%	28	7.3%	62	16.2%	48	12.6%	39	10.2%
Concern about own appearance	I am worried about the fact that I shall not regain my figure after delivery.	75	19.6%	28	7.3%	97	25.4%	110	28.8%	72	18.8%
	I am concerned about my unattractive appearance.	141	36.9%	33	8.6%	93	24.3%	80	20.9%	35	9.2%
	I am worried about my enormous weight gain.	107	28.0%	38	9.9%	80	20.9%	92	24.1%	65	17.0%

Table 4 summarizes the three subscale scores. The mean scores for fear of giving birth, worries about bearing a handicapped child, and concern about own appearance were 10.6 (70.7%), 6.4 (53.3%), and 8.7 (58%), respectively. The mean anxiety score was 28 out of 50 (56%).

**Table 4:** Pregnancy-related anxiety among pregnant females, Al-Ahsa, Saudi Arabia.

Subscale	Range	Mean	SD	Score %
Fear of giving birth	3-15	10.6	3.0	70.7%
Worries about bearing a handicapped child	4-12	6.4	2.4	53.3%
Concern about own appearance	3-15	8.7	3.5	58.0%
Overall score	10-50	28.0	9.2	56.0%

**Figure 1:**
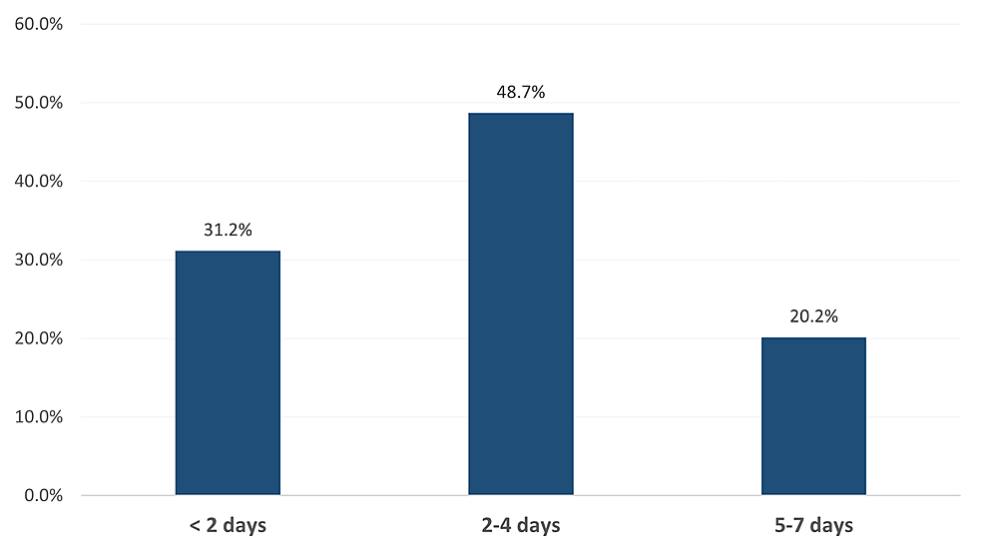
Social media engagement among pregnant females per week, Al-Ahsa, Saudi Arabia.

Figure 1 illustrates the SMEQ results of these pregnant women. Most of them (119, 31.2%) spent less than two days per week on social media, followed by two to four days per week (186, 48.7%) and five to seven days per week (77, 20.2%).

The factors associated with pregnancy-related anxiety among these pregnant women are presented in Table 5. The anxiety score was significantly higher among those working as healthcare workers (31.7) than as housewives (27.5) and non-healthcare workers (27.4) (P = 0.016). Those in their first trimester had a higher mean anxiety score than those in their third trimester (29.8 vs. 26.3; P = 0.012). Pregnant women with high adherence to ANC visits had significantly higher anxiety scores than those who never adhered (30.5 vs. 22.0; P = 0.001). The anxiety score was also higher in those with unplanned pregnancies than in those with planned pregnancies (29.2 vs. 26.9; P = 0.016). Other factors were insignificantly associated with pregnancy-related anxiety levels.

**Table 5:** Factors associated with pregnancy-related anxiety among pregnant females, Al-Ahsa, Saudi Arabia. P: one-way ANOVA; $: independent t-test; * P < 0.05 (significant).

Factors	Anxiety score		P-value
	Mean	SD	
Age in years			.650
18-25	27.1	9.1	
26-30	28.0	8.7	
31-39	28.4	10.1	
40+	29.7	7.2	
Educational level			.727^$^
Below university	27.8	9.0	
University/above	28.1	9.4	
Job title			.016*
Housewife	27.5	8.8	
Non-healthcare worker	27.4	10.4	
Healthcare worker	31.7	9.3	
Monthly income			.491
<5,000 SR	28.1	8.7	
5,000-10,000 SR	27.6	9.7	
10,000-20,000 SR	29.3	8.4	
>20,000 SR	25.6	9.8	
Is this being your first pregnancy?			.827^$^
Yes	28.2	10.1	
No	27.9	9.0	
Gestational age of the pregnancy			.012*
First trimester (1-13 weeks)	29.8	10.0	
Second trimester (14-26 weeks)	28.3	9.3	
Third trimester (27-40 weeks)	26.3	8.3	
Do you adhere to your appointments in antenatal care?			.001*
Always	26.8	9.2	
Often	30.5	8.8	
Sometimes	31.5	8.6	
Never	22.0	0.0	
Do you have complications in your current pregnancy?			.415^$^
Yes	27.2	7.8	
No	28.1	9.5	
Is this current pregnancy previously planned?			.016*^,$^
Yes	26.9	9.5	
No	29.2	8.7	
Have you ever had any complications during pregnancy or childbirth?			.580^$^
Yes	28.3	9.3	
No	27.7	8.8	
Have you ever had a miscarriage or stillbirth?			.553^$^
Yes	27.5	9.9	
No	28.1	8.5	
Have you ever had congenital anomalies?			.190^$^
Yes	30.5	8.2	
No	27.7	9.0	
Social media engagement			.550
<2	28.5	10.2	
2-4	27.45	8.3	
5-7	28.5	9.9	

**Figure 2:**
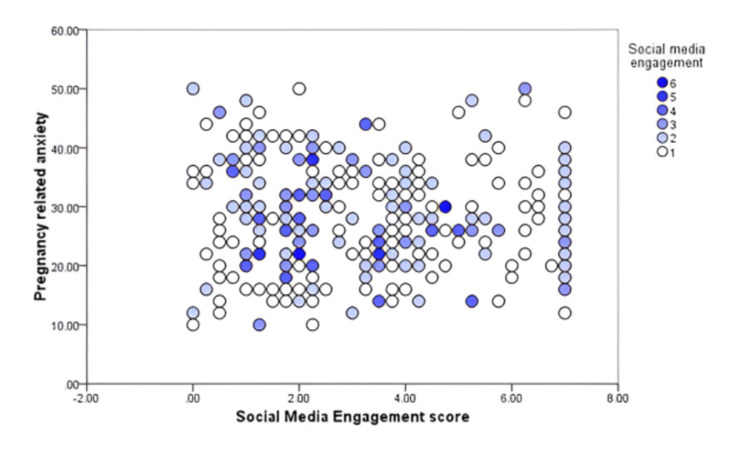
Social media engagement among pregnant females per week, Al-Ahsa, Saudi Arabia.

Figure 2 presents the correlation between pregnancy-related anxiety scores and average days of social media engagement among these pregnant women. As shown in the scatter diagram, their pregnancy-related anxiety had no significant correlation with their average use of social media per weekdays (r = 0.12; P = 0.341).

## Discussion

This study assessed the pregnancy-related anxiety and the level of extended social media use in pregnant women living in Al-Ahsa, the eastern province of Saudi Arabia, and identified the factors associated with pregnancy-related anxiety. Pregnancy-related anxiety disorders are fairly common, with nearly 10%-15% of all pregnant women feeling some level of anxiety or stress during this major transitional phase in their lives [23]. The source of pregnant women's worry is future labor and expected pain in addition to fear of childbirth [24], worry about the health of the anticipated child, or the pregnancy-associated physical changes [25]. These stressors during pregnancy can negatively affect mothers’ health and psychological well-being [26] as well as their future child. High pregnancy-related anxiety may end with unwanted pregnancy outcomes (preterm birth and low birth weight) [27].

In this study, the pregnant women generally had an average level of anxiety, obtaining a mean anxiety score of 28 out of 50 (56%). Fear of giving birth obtained the highest level of anxiety. These women were worried about the pain of contractions and delivery and about the delivery process itself. The second item with the highest anxiety score was pregnant women’s concern about their appearance. Pregnancy occurs with physiological and physical changes; thus, they were worried that they might not be able to regain their pre-pregnancy figure after delivery and might gain in weight enormously. The third was their worry about bearing a handicapped child. They were thinking that the child would be in poor health or prone to illnesses, afraid that the baby would suffer from a physical defect or something would be physically wrong with the baby, afraid that their baby would be stillborn or would die during or immediately after delivery, and afraid that the baby would be mentally handicapped or would suffer from brain damage. One of the factors that were significantly associated with high pregnancy-related anxiety in these pregnant women was being healthcare workers, which showed the highest level of anxiety because they are knowledgeable about delivery-related pain and difficulties and newborn risk of having congenital disorders or physical health problems. The second factor was early pregnancy. Pregnancy-related anxiety level was higher among women in their first trimester who were still unable to cope with pregnancy-related physical and psychological changes. The third factor was adherence to ANC visits; pregnant women with high anxiety levels showed higher adherence than those with low anxiety levels. The fourth factor was an unplanned pregnancy. Females with unplanned pregnancies showed higher levels of anxiety because such pregnancy was mostly unwanted. Perhaps, a family history of bad pregnancy outcomes or other family-related factors made them under stress for the upcoming baby.

These findings are concordant with many literature conclusions. Bayrampour et al. [28] studied pregnancy-related anxiety through concept analysis and concluded that the most frequently reported dimensions included anxiety about fetal health, fetal loss, childbirth, parenting, and newborn care. In India, Nath et al. [17] reported a similar level of pregnancy-related anxiety among pregnant females where 55.7% manifested pregnancy-related anxiety. Lower socioeconomic status, low social support, and depression emerged as significant determinants of anxiety. In a prospective cohort study, Madhavanprabhakaran et al. [29] found that a U-pattern structure of pregnancy-specific anxiety was noticed all over trimesters of pregnancy, with nulliparous childbearing women showing the highest anxiety level. Adverse delivery outcomes such as prolonged labor, preterm labor, low birth weight, and unplanned cesarean section were associated with high pregnancy-related anxiety. A systematic review conducted by Jha et al. in 2018 [30] estimated the prevalence of anxiety during pregnancy at 1%-26% in low- and middle-income countries. However, published literature on anxiety during pregnancy seems to be limited. This highly variable range may be attributed to many factors, including the scale used to assess the anxiety level, phase of pregnancy at which the study was conducted, related health problems, and history of psychological disorders.

Considering social media engagement, the current study showed that roughly half of the pregnant females engaged in social media for two to four days per week, whereas only one-fifth (20.2%) may use social media for five to seven days per week. Despite the high engagement, no significant association was found between the social media engagement rate and pregnant women’s level of anxiety.

## Conclusions

Pregnant women in the eastern province of Saudi Arabia showed an average level of pregnancy-related anxiety, which was mostly caused by fear of childbirth and concern about their appearance. Anxiety was significantly associated with early pregnancy, being healthcare workers (who were knowledgeable about pregnancy and newborn care), unplanned pregnancy, and adherence to ANC visits. In addition, their social media engagement rate was high but was not significantly associated with their anxiety level. Early assessment and detection, prevention, and management of pregnancy-related anxiety are necessary to enable women to cope with the challenges of pregnancy and the related changes.
